# Electrostatically Driven In-Plane Silicon Micropump for Modular Configuration

**DOI:** 10.3390/mi9040190

**Published:** 2018-04-18

**Authors:** Sebastian Uhlig, Matthieu Gaudet, Sergiu Langa, Klaus Schimmanz, Holger Conrad, Bert Kaiser, Harald Schenk

**Affiliations:** 1 Fraunhofer Institute for Photonic Microsystems, IPMS, 01109 Dresden, Germany; matthieu.gaudet@ipms.fraunhofer.de (M.G.); sergiu.langa@ipms.fraunhofer.de (S.L.); holger.conrad@ipms.fraunhofer.de (H.C.); bert.kaiser@ipms.fraunhofer.de (B.K.); harald.schenk@ipms.fraunhofer.de (H.S.); 2 Fraunhofer Institute for Photonic Microsystems, IPMS, 03046 Cottbus, Germany; 3 Chair of Micro and Nano Systems, Brandenburg University of Technology Cottbus-Senftenberg, BTU C-S, 03046 Cottbus, Germany; klaus.schimmanz@ipms-extern.fraunhofer.de

**Keywords:** micropump, electrostatic actuation, nano e-drive, in-plane reciprocating displacement micropump, MEMS

## Abstract

In this paper, an in-plane reciprocating displacement micropump for liquids and gases which is actuated by a new class of electrostatic bending actuators is reported. The so-called “Nano Electrostatic Drive” is capable of deflecting beyond the electrode gap distance, enabling large generated forces and deflections. Depending on the requirements of the targeted system, the micropump can be modularly designed to meet the specified differential pressures and flow rates by a serial and parallel arrangement of equally working pumping base units. Two selected, medium specific micropump test structure devices for pumping air and isopropanol were designed and investigated. An analytical approach of the driving unit is presented and two-way Fluid-Structure Interaction (FSI) simulations of the micropump were carried out to determine the dynamic behavior. The simulation showed that the test structure device designed for air expected to overcome a total differential pressure of 130 kPa and deliver a flow rate of 0.11 sccm at a 265 Hz driving frequency. The isopropanol design is expected to generate 210 kPa and pump 0.01 sccm at 21 Hz. The device is monolithically fabricated by CMOS-compatible bulk micromachining processes under the use of standard materials only, such as crystalline silicon, silicon dioxide and alumina.

## 1. Introduction

In many application fields, such as microchannel-cooling [1,2], miniaturized chemical analysis [3,4], gas-chromatography [5,6], and mobile on-chip applications [7,8], there is a need for a microscale pumping device that exhibits, besides a specific flow rate and pressure difference, low power consumption and a reduced device size. Electrostatic-driven silicon micropumps are a part of Micro-ElectroMechanical Systems (MEMS) and can often meet these demands [9,10,11]. Their advantages in terms of scalability of the drive mechanism, simplicity in their fabrication, low power consumption, and fast response time, make them an attractive choice for many applications. The majority of these devices function with an out-of-plane mechanism of a mechanically reciprocating membrane clamped at its perimeter. Here, the challenge is to precisely design the diaphragm and the drive, in order to reach a sufficient stroke volume, i.e., a compression ratio [12,13], for the pumping effect and a failure-free operation. The small deflections of micro-actuators further complicate this task. For common electrostatic actuation mechanisms (e.g., plate capacitor geometry), this is especially challenging, due to the dependence of the maximum deflection on the electrostatic gap, and more importantly, the limiting “pull-in” effect [14]. 

With the concept of the Nano Electrostatic Drive (NED) driving principle, presented by our group in 2015 [15], a novel class of electrostatic silicon bending actuators was introduced. The NED offers a new approach in designing electrostatically actuated micropumps. The micropump presented in this paper utilizes an in-plane NED variant as the active driving mechanism [16]. NED actuators commonly comprise a series of electrodes with a defined shape, separated by an electrostatic gap in the range of a few micrometer, and insulating spacer islands. Upon applying a control voltage +*V* to the electrodes, the electrostatic forces are transferred into lateral mechanical forces. The result is a tip displacement greater than the initial electrostatic gap separation and a cylindrically curved bending of the actuator [15,16,17,18]. The actuation of NED beam actuators in a liquid environment was already experimentally investigated [18]. In terms of generated force and tip displacement, an increase determined by the relative permittivity of the dielectric liquid was reported. In doing so, the actuator was fully immersed in the liquid, and the electrostatic gaps were completely filled. 

By using a NED actuator as moving wall and by combining this with rectifying in-plane check valves, a reciprocating pumping effect can be achieved. The thin beam-like structure of the actuator beam allows to stack it in an efficient and spatially compact way throughout the chip. This opens the possibility of modularly designing a micropump that can meet application specific flow rates as well as differential pressure needs for pumping liquids and gases. As a result, the performance of the micropump stays comparable to existing high performing solutions (e.g., [8,9,10,11]), by being able to provide flow rates in the range of tens of µL/min and withstanding backpressure of hundreds of kPa. Furthermore, only standard complementary metal-oxide-semiconductor (CMOS) compatible fabrication processes and materials are used to monolithically fabricate the device [16,17], without the need of a hybrid assembly of passive and active pump components.

In this paper, two medium specific micropump test structure designs are presented. Their development from an analytical understanding of the actuation unit up to the detailed description of the simulation setup and the fabrication process is given in Section 2. The simulation results and expected performance parameters are presented and discussed in Section 3.

## 2. Materials and Methods

### 2.1. Actuation Theory of the In-Plane Micropump

**Figure 1:**
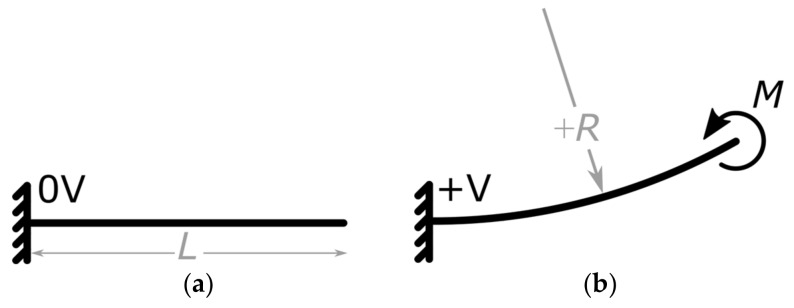
Representation of clamped-free NED actuator with length *L*. Radius of curvature defined positive, as shown. (**a**) Non-actuated state; (**b**) actuated state—cylindrical bending line.

When the NED cantilever is arranged in a clamped-free (c-f) configuration, it deforms cylindrically, with a curvature C, upon actuation [15]. Following this, the actuator is approximated by a solid cantilever beam, which, under the Euler Bernoulli hypothesis, is associated with small deflections [19], as shown in Figure 1. In an actuated state, the cantilever beam exhibits a curvature  + R = 1/C, generating a constant bending moment M along the beam. 

The bending moment thereby depends on the geometrical and material parameters of the NED actuator beam, as well as on the applied control voltage and dielectric permittivity of the fluid between the electrodes [15,18]. By interconnecting four NED actuator beams and arranging them to a clamped-clamped (c-c) configuration under the use of an alternating radius scheme (+R, −R, −R, +R) the effective bending Moment $M_{eff}$ is increased to 2M. This can be associated to mechanical moments applied at $¼\;L{}^{\prime }$ and$\;¾\;L{}^{\prime }$, compared with the (c-f) case in Figure 1a, where M applied at L. The total length in (c-c) configuration adds up to$\;L{}^{\prime }\;=\;4L$.

The maximum deflection $\delta_{max}$ occurs at the center of the clamped-clamped beam and can be derived for the case of pure bending (constant moment, i.e., no shear-forces) to:

(1) $$\delta_{max}=\frac{M_{eff}\;L^{\prime 2}\;}{32\;EI}$$

**Figure 2:**
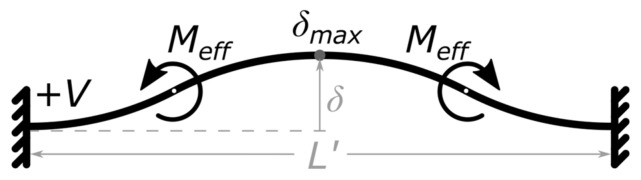
Sketch of four specifically arranged NED actuator beams and their bending line in (c-c) configuration, when actuated.

Here, EI is the equivalent bending stiffness of the physical actuator. The advantage of this configuration is that the two sides of the NED actuator beams (meaning schematically above and below the sketched actuator beam bending line in Figure 2), are fluidically separated and can act as force-applying moving walls, generating a volume variation during the operation. The stroke volume ΔV is the area passed over by the actuator beam multiplied by its height h (in Figure 2 perpendicular to the paper plane):

(2) $$\Delta V=\left(\frac{L^{\prime }}{2}\;\delta_{max}\right)\;h\;$$

**Figure 3:**
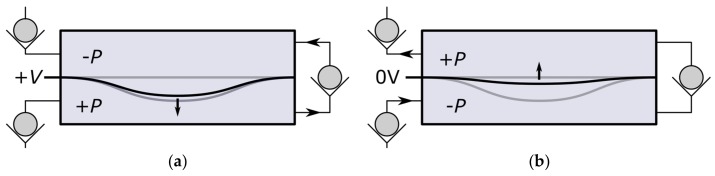
Sketch of the pumping principle based on a NED actuator beam in (c-c) configuration in combination with 3 passive check valves: (**a**) Active displacement associated to a pump stroke; (**b**) Passive displacement associated to an intake stroke. Flow and actuator movement direction indicated by arrows.

By placing one NED actuator beam in (c-c) configuration with both sides fluidically separated by three ideally rectifying check-valves, a simple pumping effect can be achieved. This situation is shown in Figure 3. Assuming an ideal incompressible fluid, a gauge overpressure +P is created in the cavity compressed by the actuator, while an associated gauge underpressure −P, is created in the expanded cavity (Figure 3a). This gives a total differential pressure of twice the absolute gauge pressure ΔP=(P−(−P))=2P, between the two cavities, resulting in a pressure-driven flow.

The maximum generated pressure difference$\;\Delta P_{max}$, during this operation, can be obtained by calculating the maximum gauge pressure in one cavity. It depends on the maximum generated force of the actuator in relation to the surface facing the fluid of the cavity. The so called “blocking force” $F_{BL}\left(\delta =0\right)$ is achieved when at applied voltage +*V* the displacement is completely blocked [20]. It is calculated by setting the maximum deflection of the NED beam, given by Equation (1), to the equal maximum deflection of a clamped-clamped (c-c) beam, of equal stiffness and length, under a point-load at the center:

(3) $$\delta_{\max}\left(M_{\mathrm{eff}}\right){=\delta }_{\max}\left(F\right),$$

(4) $$\frac{M_{eff}\;L^{\prime }{}^{2}\;}{32\;EI}=\frac{F\;L^{\prime }{}^{3}}{192\;EI}$$

Solving for *F* yields$\;F_{BL}:$

(5) $$F_{BL}=6\frac{M_{eff}}{L^{\prime }}$$

Equation (5) states that the actuators effective bending moment gets transferred into a point-load at the center of the beam. By relating that point-load to the area facing the fluid$\;\left(A_{Act.-Fl.}=L^{\prime }h\;\right)$, in the cavity being compressed and multiplied by two, the corresponding maximum differential pressure can be obtained:

(6) $$\Delta P_{max}=2\frac{F_{BL}}{A_{Act.-Fl.}}$$

**Figure 4:**
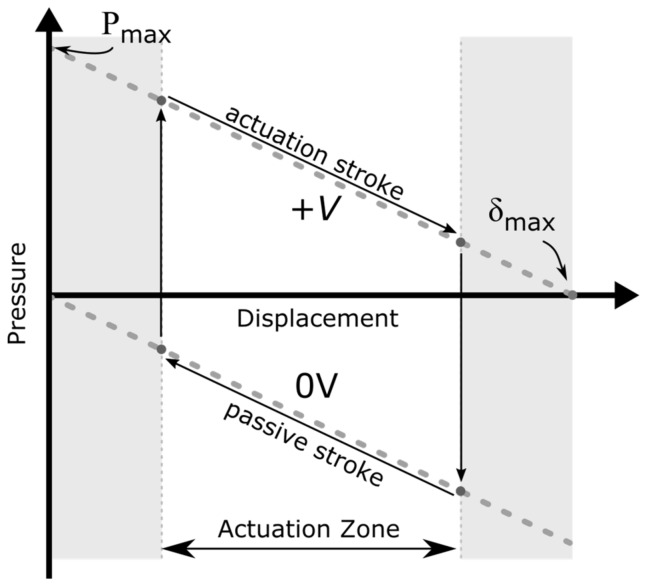
Pressure/displacement graph and concept of NED actuation scheme in the micropump.

Such kinds of bending actuators follow a characteristic actuator curve, as shown in Figure 4. At each position of the active displacement, the actuator delivers a specific gauge overpressure in the cavity being compressed, as shown in the upper part of the graph. In the same manner, canceling the applied moment, i.e., switching off the control voltage, will allow the deformed actuator to release the elastic bending energy and move back to its original position. As a result, the previously compressed cavity is expanded, generating a gauge underpressure. This is shown schematically in Figure 3b and represented in the lower part of the graph in Figure 4.

During a full cycle, both cavities (on the lower and the upper side of the beam; see Figure 3) experience a compression and a subsequent expansion, which can be associated to a pump and intake stroke, correspondingly. The resulting flow rate during the cycle thereby depends on the generated differential pressure, flow channel geometry, and fluid properties [21]. By letting the NED actuator beam displace over its full range and back to the initial position, a flow rate is to be expected which is lower than the maximum achievable flow rate of the set-up. This is due to the fact that at displacements near $\delta_{max}$ in the active stroke, and δ=0 in the passive stroke, the generated gauge pressure tends to zero. Hence, the stroke volume takes a longer time duration to be pumped. On the other hand, switching off the driving voltage at a too small displacement results in a smaller generated gauge pressure in the cavities (i.e., smaller differential pressure), and thus, unequal stroke durations. This consequently leads to a reduced output flow rate, as well. 

An optimum flow rate can be achieved if the generated gauge pressure, at the beginning of active and passive stroke is the same, resulting in equal time durations of pump and intake phase. With the exception of the extreme case of a full displacement range operation, which takes a longer stroke time, this is also the case for a reduced symmetric zone operation around the actuators’ half displacement, with significantly shorter stroke durations. Here, the shorter stroke durations also mean smaller stroke volume, but the stroke durations will have higher influence on the flow rate. For that reason, it is of interest to actuate in a defined displacement interval around a working point, chosen to be at the actuators half displacement. This is shown schematically in Figure 4. The electrical excitation of the actuator is chosen as a periodic step function rather than a sinusoidal function, in order to have the generated pressure instantly available, and not building up following the characteristic pressure/displacement graph.

### 2.2. Base Unit Design of the Micropump

**Figure 5:**
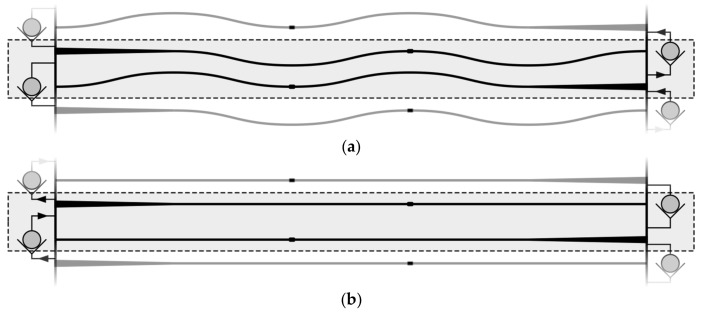
Base pumping unit of NED micropump (dashed rectangle marks off imaginary unit boarder). (**a**) Active stroke, transfer of fluid via valves on right-hand side; (**b**) Passive stroke, transfer of fluid via left-hand sided valves. Arrows indicate direction of flow.

The advantage of the NED actuator is that its maximum generated bending moment (i.e., its maximum generated differential pressure) can be optimized for a specific fluid with dielectric permittivity at a targeted control voltage. With that in mind, two micropump test structures were designed with two different actuator design points. Design point A for pumping air is operated at +300 V, and design point B for Isopropanol (IPA) is operated at +100 V. The design consists of multiple identical pumping units: the so-called “base units”. A base unit is composed of 4 NED actuators in (c-c) configuration, as displayed in Figure 2, with two actuators placed in series and another two facing each other with shift of$\;½\;L{}^{\prime }$. Figure 5 shows an illustration of a base unit with adjacent units indicated above and underneath. 

During the active stroke, the actuators inside the base unit deflect in opposite directions. Thereby, the flow channel inside the base unit is compressed, while the flow channel between two neighboring base units is expanded. The generated differential pressure by the actuators between the flow channel cavities establishes a flow from one base unit to the next through the passive valves, indicated as check valves in Figure 5. The base units are periodically stacked, which increases the total length of the flow channel. This increases the differential pressure drop for an imposed backpressure from the outlet to the inlet of the micropump. The advantage of this is that each base unit acts only as an *N*-th part of the back pressure, with *N* being the number of periodically stacked units. Thereby, the generated differential pressure is added to the fluid in each of the pump unit stages. This creates a stage-like structure of the micropump. As a result, a larger pressure difference between the inlet and outlet can be overcome.

### 2.3. Modular Configuration of the Micropump

**Figure 6:**
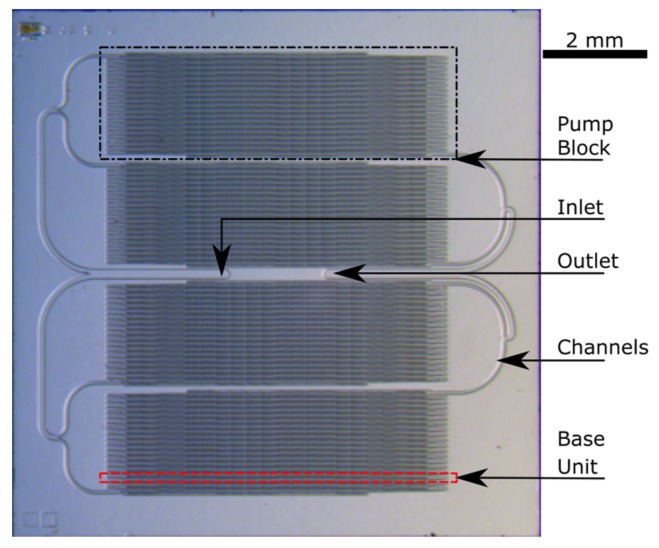
Micrograph of the device layer of the complete in-plane test structure chip dedicated to pumping IPA. The dashed rectangle indicates one base pumping unit within one pump block, while the dash-dotted rectangle indicated the pump block itself.

The approach of setting up equally working base pumping units, which contains a serial arrangement of NED actuator beams as well as passive rectification valves, opens the possibility of modularly designing a complete micropump with desired output specifications. A defined stack of base units makes up one pumping block. These blocks can further be arranged in parallel, which increases the total pumped volume, thus, the output flow rate of the micropump. The device layer of the modularly configured micropump test structure, for the design of pumping IPA, is presented in Figure 6.

It is composed of four pumping blocks, each containing 19 base units with four NED actuator beams in (c-c) configuration. Each pumping block has its own set of electrical connection from the backside of the chip. This permits the actuation of the blocks with a phase shift which is expected to reduce the output flow pulsation. The fluidic micro-channels connecting the inlet and outlet of each block to the main inlet and outlet of the micropump test structure are also visible. 

### 2.4. Micropump Test Structure Setup and Fabrication

**Figure 7:**
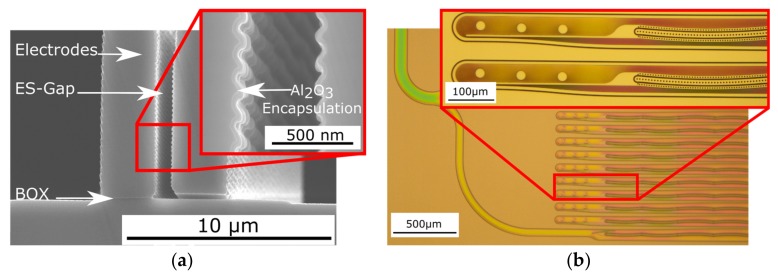
Micrographs of fabricated NED micropump test samples. (**a**) DRIE test etching of electrostatic gap (BOX-layer not released). Inset shows encapsulation of the NED electrodes by 60 nm ALD-Al_2_O_3_; (**b**) Device layer of micropump test sample designed for IPA. Inset shows passive flap valves and beginning of defined shaped NED electrodes.

The micropump test structure chip is composed of a standard wafer, which is bonded on a bonded Silicon On Insulator (SOI) wafer, with 1 µm Buried-Oxide (BOX). The bond interface between the top cover and the SOI is thermal grown Silicon-Dioxide (SiO_2_) of 1 µm as well. The active device layer has a height of 75 μm and contains the driving NED actuator beams as well as passive valves and fluidic channels. It is processed by standard bulk-micromachining technologies, such as Deep Reactive Ion Etching (DRIE). Figure 7a shows the result of a test etching of the electrostatic gap of the NED electrodes. The fluidic inlet and outlet are located on the top cover, while the electrical connections are located on the bottom cover (SOI-handle), making up a total chip thickness of approximately 900 μm. This enables an easy integration of the chip into a measurement system. The entire fabrication process is compatible with CMOS-technologies, using crystalline silicon, silicon-dioxide, and aluminumoxid only.

A micrograph of a processed device layer of the micropump test structure is presented in Figure 7b, where the feed channels from the inlet and the left part of the periodically arranged pumping base units are shown. The inset shows the magnified passive valves of two base units and the beginning of the defined shaped electrodes of the NED electrostatic-bending actuators. The currently fabricated design points of the micropump test structures are chosen to test the working principle with non-conductive fluids. However, in terms of perspective, the intention is to pump conductive fluids, as well. In order to avoid electrical short-circuiting, the electrodes are additionally encapsulated by either a layer of 60 nm Al_2_O_3_ (Figure 7a), deposited by Atomic Layer Deposition (ALD), or in another split by a 200 nm layer of Parylene. 

**Figure 8:**
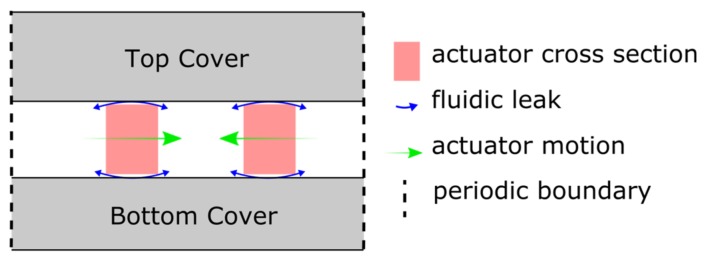
Sketch of a cross-section through the micropump test structure chip shows the sandwich of the three-layer silicon wafer stack. The NED actuators of one base unit moving in plane (indicated). The fluidic leak through clearance above and below the actuators is shown as well.

Figure 8 shows a sketch of a cross-section of a fabricated wafer stack going through the middle of a base unit. To ensure that the actuators and the valves can move laterally in the wafer stack, the 1 µm oxide layer is locally removed. The remaining clearance presents a fluidic leak that is small compared to the fluid transfer in the channels (cf. Figure 7b). It manifests itself as a dead volume during the operation. The additional encapsulation of the actuators by Parylene or Al_2_O_3_ further reduces this clearance. 

### 2.5. Simulation Setup of the Micropump

**Figure 9:**
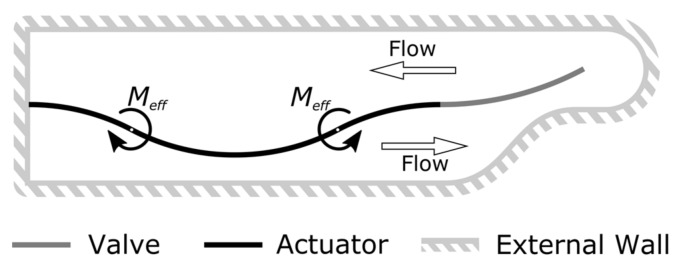
Sketch (2D) of the model setup used for the 2-way FSI simulation of the behavior of one base unit.

In order to validate the concept and the behavior of the NED in-plane micropump test structures, a transient two-way Fluid-Structure Interaction (FSI) simulation [22] using an ANSYS Workbench was carried out. The multiphysics problem is thereby treated in a partitioned approach. This means that the governing equations of the structural and fluid domain are solved separately, and their resulting data (i.e., displacement and pressure, respectively) is exchanged in a coupling application between the individual solvers. This was achieved by combining the toolboxes “transient mechanical” and “FLUENT” via the “system coupling” toolbox, which controlled the data transfer and the number of coupling steps used. The two-way FSI simulation therefore takes into account the effect of the structural domain on the fluid, as well as the subsequent reaction of the fluid on the structural part. To reduce the computational complexity, a simplified model of the described base unit was created. The FSI model consists of a cavity of one NED actuator beam in (c-c) configuration of length $L{}^{\prime }$ and one passive flap valve, with the same dimension as the designed base unit of the micropump test structures. This model is sufficient to cover the behavior of pumping the fluid through the structure of the micropump to the outlet, since each stage works equivalently and transfers the fluid from underneath to above the actuator through the separating passive valve. The clearance was not considered in this model. A 2D sketch of the FSI model’s geometry is shown in Figure 9.

Because of the anisotropic DRIE fabrication process, the device layer of the micropump can be seen as an extrusion of the 2D geometry into the 3rd dimension. Thus, only a thin 2.5D slice, (extrusion of 2D geometry into 3D) of one finite-volume height of 4 µm was simulated, instead of the entire 3D structure of the pump. This simplification further reduces the computational complexity. As a result, in this model the choice of a 4 µm 2.5D-slice height ensured finite volumes with a high mesh quality (skewness of 0.6, orthogonal quality of 0.7). The top and bottom plane of the 2.5D model slice (the paper plane in Figure 9) were set up as symmetric boundary conditions, consequently neglecting the influence of the top and bottom walls of the fluid channel. This approach is valid due to the high aspect ratio of channel height and channel width of more than 15, as can be seen in Figure 7b [23].

**Figure 10:**
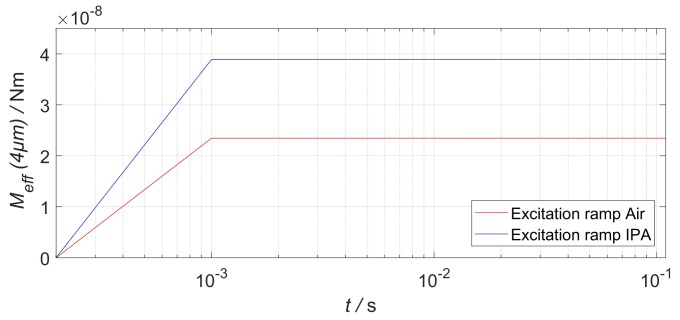
Ramped step function of NED actuator beam effective moments in “ANSYS mechanical”. The values are scaled to a 2.5D model slice height of 4 µm.

In the structural domain, the entities’ actuator and valve were meshed using higher-order SOLID186 elements. The actuator itself was modelled as a solid beam, with equivalent bending stiffness EI, and equal footprint of the physical NED actuator beam. The effective moments $M_{eff}$ were scaled to the 2.5D model height and applied at the positions of $¼\;L{}^{\prime }$ and$\;¾\;L{}^{\prime }$ via a step excitation. In order to ensure convergence of the model during the pressure buildup, the moment step was ramped with a time constant of $t_{ramp}=10^{-3}\;$s, as shown in Figure 10. The generated pressure of the 2.5D slice model remains the same as for a full 3D structure, since the force (i.e., applied beam moment) and area facing the fluid is scaled down equivalently. Further boundary conditions in “ANSYS mechanical” included the fixed supports and the fluid–solid interfaces (actuator and valves sides facing the fluid). During the simulation, the fluid reaction data is mapped as a load onto these interfaces. Additionally, nonlinear stiffening effects were considered in the structural model.

The fluid domain was setup in “FLUENT”. For lengths scales >100 nm, the fluid can be considered as a continuum of fluid particles, meaning that no significant fluctuation of the viscosity is expected in the physical case [23,24]. The viscosity and density values used in the simulation are based on a reference temperature of 288.15 K. The small microchannel dimensions in the geometry (channel widths ranging from 3–30 µm) and a differential pressure drop in the kPa range generate flow velocities of a few m/s, leading to low Reynolds numbers (Re≈100). At values lower than 2300, the flow can be considered as laminar in the system. Consequently, the model for laminar flows with constant viscosity in “FLUENT” was selected. In the material properties, air was modelled as ideal gas, while IPA was modelled as a compressible fluid, with a maximum allowable change in density of ±2%. This is of high importance, because in a closed volume system with deforming boundaries, as modelled above, a constant density would imply an infinite wave speed and result in a non-converging model [25]. Because of large structural deformations (i.e., valve and actuator deflection), relative to the finite-volume mesh size, the option deforming mesh in combination with mesh smoothing was applied. For the remeshing, the 2.5D option was selected, while the mesh smoothing was based on the spring-layer boundary method.

The computational fluid dynamic (CFD) solution was obtained by using the pressure-based solver with the SIMPLE algorithm. Furthermore, the solution was stabilized by using the coefficient-based stabilization factors with the declared fluid–solid interfaces.

The time step used in the FSI simulation was declared in the “system coupling” toolbox, as well as in the “mechanical” toolbox, and was chosen to reach a proper convergence of the flow field and data transfers between the solvers in the “mechanical” and “FLUENT” toolboxes. A complete list of solver settings and convergence criteria is given in Appendix A of Table A1, while an error analysis is presented in Appendix B. The corresponding actuator and valve design point values used in the simulation are summarized in Table 1.

## 3. Results and Discussion

### 3.1. Simulations Results

**Figure 11:**
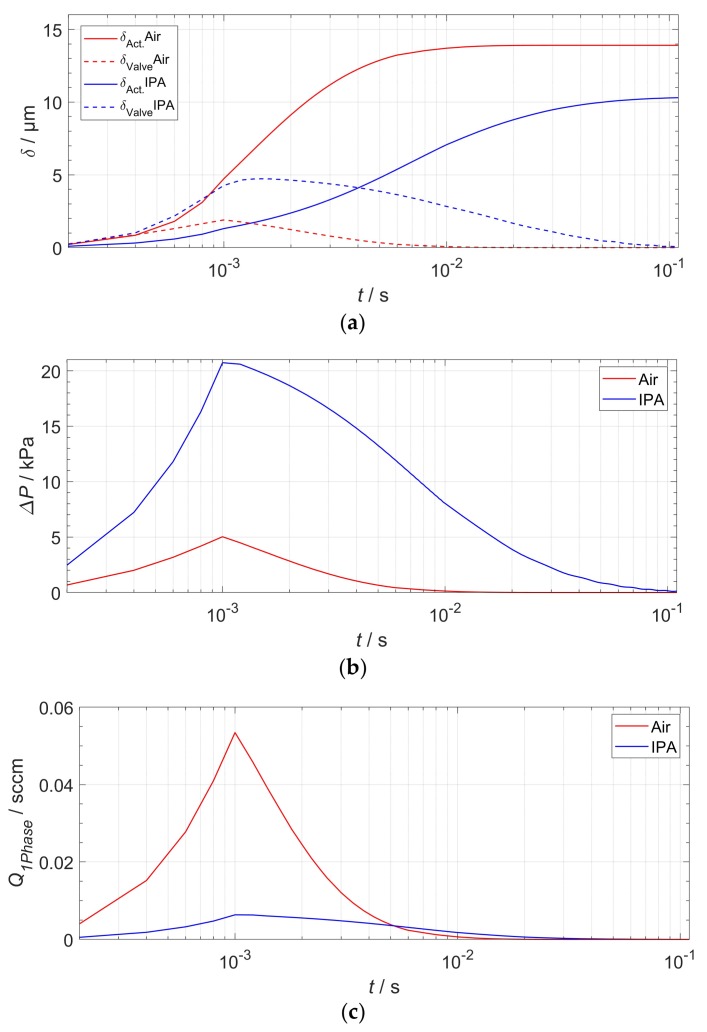
Results of the transient FSI simulation. (**a**) Maximum deflection of actuators and valves; (**b**) Differential pressure behavior; (**c**) One-phase flow rate through the cavity extrapolated to 75 µm of the device layer height.

The transient FSI simulation was carried out over the active stroke: half a full pump cycle until a total end time of$\;t_{end}=10^{-1}$ s. For each time step$\;\Delta t_{step}$, the actuator deflection in the center (cf. Figure 2); the tip deflection of the passive valve, the flow rate, as well as the maximum pressure difference between the two actuator sides were monitored. At the same time, the flow rate was read out at a cross-sectional face localized at the position of the indicated upper arrow in Figure 9. The readout process by the software included the calculation of the integral of flow velocities across that face. The simulation results are plotted in Figure 11.

**Figure 12:**
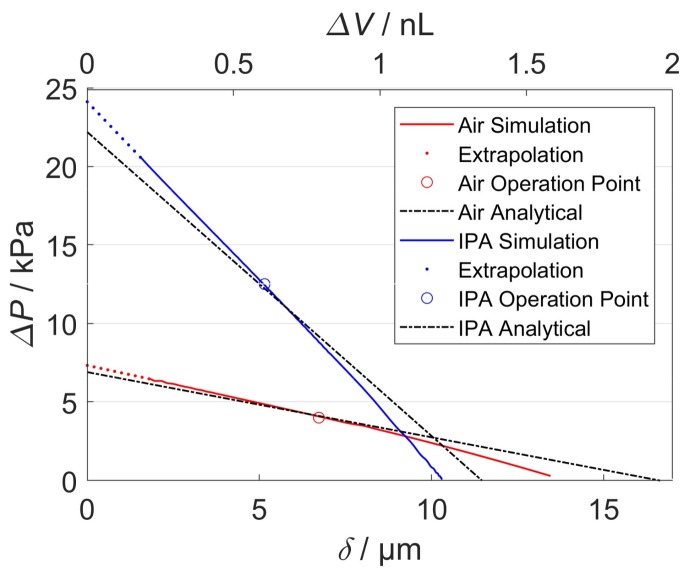
Generated differential pressure vs. displacement graph, obtained from the simulation results as well as analytical model. The resulting stroke volume for corresponding displacements of the NED actuator beams in (c-c) configuration is written on the top *x* axis. The intended operation point of the micropump is at the half of the actuator displacement.

Figure 11a shows the transient displacement behavior of the two NED actuator beams and valves in the corresponding test medium air and IPA. The actuator of the pump designed for air reaches its maximum deflection of $\delta_{max}\left(air\right)=13.5\;\text{µ}m$ about one temporal order of magnitude earlier than in the case of IPA, $\delta_{max}\left(IPA\right)=10.3\;\text{µ}m$. The different damping response behavior arises mainly from the effect of the viscosity and density of the two different media. After the maximum moment is reached at $t_{ramp}=10^{-3}$ s, the established differential pressure Δ*P* and flow rate (Figure 11c) decrease asymptotically to zero. This can be explained by the damped displacement of the actuator (cf. Figure 11a) and the pressure–displacement behavior, as described above (cf. Figure 4), whereby less gauge pressure is generated by the actuator in the cavities at larger displacements. Because the NED actuator beam designed for IPA generates a higher differential pressure than the designated point of air, the corresponding mechanical load and valve tip deflection is also higher. By plotting the data of the differential pressure (Figure 11b) against the actuator displacement (Figure 11a), one can once again find the generated pressure–displacement relation of the NED actuator beams, as shown in Figure 12. Only the simulation data from the time after the full moment was applied ($t>t_{ramp}$) were used for the plot. The graphs show a linear relation for high differential pressure values (e.g., small displacements), as expected. For large displacements near the maximum, the data deviates from a linear behavior, due to nonlinear mechanical stiffening effects considered in the structural domain.

The maximum differential pressure, associated to a fully blocked deflection (δ = 0 µm) of the actuator, was obtained by a linear extrapolation (dotted) of the simulated data at lower displacements. For the IPA design point, the actuator delivers $\Delta P_{max}=24.1$ kPa, while the actuator for air generates $\Delta P_{max}=7.2$ kPa because of a lower effective moment and a larger actuator length. A comparison of the FSI simulation to the simplified analytical approach reflects the expected behavior of the system. The deviations of the maximum differential pressure to the simulation data can be accounted to the “blocking force” approach described in the previous section (Equation (5)) as well as uncertainty from linear extrapolation of the data.

### 3.2. Actuation Zone and Dynamic Pump Behavior

To investigate the effect of an actuation interval on the total output flow rate, that is, the flow rate of the pump cavity after a full cycle of active and passive stroke, the simulation data shown in Figure 11a,c was further analyzed. A symmetric actuation zone around the operation point, set at the half deflection of the actuator, ensures equal duration of both strokes. Therefore, the displaced volume during the active stroke can be divided by twice the stroke duration for the full cycle output flow rate. For a defined interval, such as the displacement width$\;\Delta \delta =\delta_{2}\left(t_{2}\right)-\delta_{1}\left(t_{1}\right)$, the pump’s displaced volume is the actuator stroke volume of this interval minus the dead volume of the valve at the end of the interval. The dead volume of the valve was calculated from the tip displacement (Figure 11a) and total valve length (Table 1) by:

(7) $$V_{dead}\left(t_{2}\right)=\frac{L_{valve}}{2}\delta_{valve}\left(t_{2}\right)$$

The total output flow rate is determined by:

(8) $$Q_{total}\left(\Delta \delta \right)=\frac{\Delta V\left(\Delta \delta \right)-V_{dead}\left(t_{2}\right)}{2\Delta t\left(\Delta \delta \right)}$$

**Figure 13:**
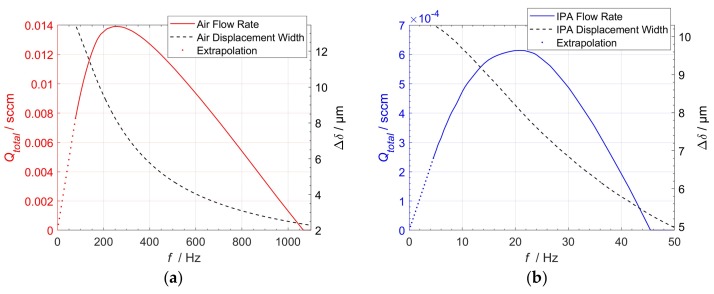
Frequency-dependent output flow rate behavior for (**a**) Base unit Design A (air); (**b**) Base unit Design B (IPA). The dashed curves (left *y* axis) display the corresponding actuation zone widths. (Dotted) Linear Extrapolation to zero flow rates due to simulation end time.

The resulting total flow rates, depending on the periodic step actuation frequency (f=1/(2Δt)) for both design points, are given in Figure 13. For pumping air, the maximum output flow rate peaks at 0.0139 sccm for 265 Hz. This corresponds to an actuation zone width of 7.9 µm. In the case of pumping IPA, the maximum total flow rate occurs at 0.62 × 10^−3^ sccm at 21 Hz, for an actuation zone width of 8 µm. The maximum displacement width of each actuator is limited by the corresponding maximum deflection$\;\delta_{max}$. Therefore, only the flow rate data up until this limit were obtained by this analysis. For flow rates in the low frequency range, that is, the actuator staying at maximum deflection, the simulation data were linearly extrapolated until zero flow rates. 

### 3.3. Extrapolation of Simulation Results to the Designed Micropump Test Structure Chips

Because of the equal working behavior of each actuator beam and thus, each base pumping unit, the obtained simulation results can be linearly extrapolated from the simulation setup of one actuator and valve to the complete micropump test structure arrangement. Each NED actuator beam inside a base unit therefore generates a certain stroke volume $\Delta V_{total}\;\left(\Delta \delta \right)$ depending on the calculated displacement width. Since one base unit is made up of four NED actuator beams, the total stroke volume per base unit is$\;4\Delta V_{total}$. The total operational differential pressure generated by each micropump test design is the differential pressure at the defined operation point of each base unit times the number of stacked base units per block. Each block then pumps the total stroke volume of one base unit. However, the parallel arrangement of these pumping block increases the total stroke volume and hence the total output flow rate by the number of blocks. The resulting specifications and output parameters of both test structure chip design points are given in Table 2.

## 4. Conclusions

In this paper, we present a novel approach of an in-plane driven silicon micropump which is actuated electrostatically and capable of pumping liquids and gases. A laterally deflecting variant of the NED was used in a clamped-clamped configuration, acting as a moving wall on the fluid. The rectifying passive flap valves work in-plane as well. Two medium specific micropump test structures for pumping air (Design A) and isopropanol (IPA) (Design B) have been designed and simulated to prove the working principle with non-conductive fluids.

The driving NED actuator beams were optimized on a maximum bending moment, in the specific test medium, to be operated at a voltage of +300 V for design A and +100 V for design B. Previous works on the NED showed the strong dependency of the tip deflection on the relative permittivity of the liquid it is actuated in. With the help of a two-way FSI simulation, it was shown that the expected output flow rate of design A is 0.11 sccm for a 265 Hz periodic step function excitation. The maximum expected output flow rate of design B is 0.01 sccm at 21 Hz. This maximum output flow rate can be achieved by a proposed actuation of the NED in a certain displacement interval at around the half maximum deflection of the actuator. Design A was expected to deliver a maximum differential pressure of 130 kPa, while for design B, 210 kPa was expected. The analytical development of the driving unit is in close agreement with the simulation results. Each test structure design exhibits a modular configuration of equally working base pumping units that are linearly and periodically arranged (increasing the operational differential pressure) and in parallel to increase to pumped volume, thus output flow rate.

The modular configurable design of the proposed electrostatic micropump opens the possibility of targeting application-specific differential pressures and flow rates, in an efficient chip area saving manner. Furthermore, market economic aspects, such as compatibility and low production cost, can be met by the monolithic fabrication of the device, using only standard microsystem materials and technologies.

## Figures and Tables

**Table 1:** NED actuator design point and fluid data used for two-way FSI simulation.

Design	A	B
Medium	Air	IPA
$\varrho_{fluid}\;\left(288.15\;K\right)$	1.225 kg/m³	790 kg/m³
$\eta_{fluid\;}\left(288.15\;K\right)$	1.79 × 10^−5^ Pas	0.0012 Pas
EI	8.44 × 10^−9^ Nm²	1.05 × 10^−8^ Nm²
EI (4 µm)	4.49 × 10^−10^ Nm²	5.58 × 10^−10^ Nm²
$M_{eff}$	2.20 × 10^−7^ Nm	3.65 × 10^−7^ Nm
$M_{eff}\;\left(4\;µm\right)$	2.35 × 10^−8^ Nm	3.89 × 10^−8^ Nm
$L{}^{\prime }$	3.2 mm	2.3 mm
$L_{valve}$	0.3 mm	0.3 mm
$t_{ramp}$	10^−3^ s	10^−3^ s
$\Delta t_{step}$	10^−4^ s	10^−4^ s
$t_{end}\;$	10^−1^ s	10^−1^ s

**Table 2:** Specification and estimated output parameters of in-plane micropump designs based on extrapolated simulation results.

Design	A	B
Medium	Air	IPA
Size	10 × 7 × 0.9 mm³	10 × 10 × 0.9 mm³
Actuation voltage	300 V	100 V
# of parallel blocks	2	4
# of base units per block	19	19
# of NED actuators per base unit	4	4
Operational differential pressure	130 kPa	210 kPa
Expected flow rate	0.11 sccm	0.01 sccm
Operational actuation frequency	265 Hz	21 Hz

**Figure A1:**
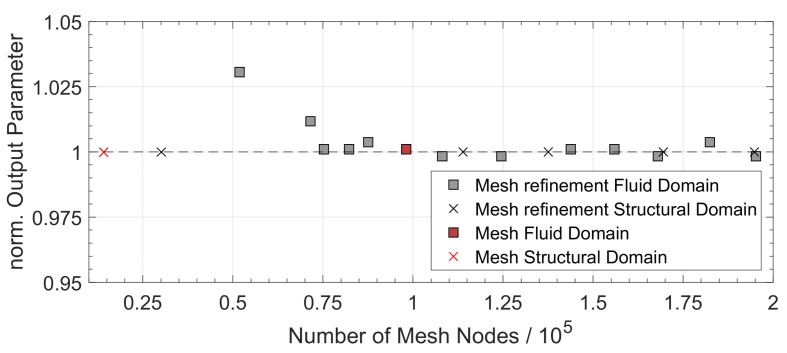
Results of the spatial discretization refinement analysis of the structural and fluid domain mesh (grid). The chosen mesh densities used in the simulation are indicated in red.

## Appendix A

**Table A1:** Summary of solver settings in ANSYS Workbench used in the two-way FSI Simulation.

Toolbox	Property	Setup/Value
Transient Mechanical	Solver	Program Controlled
	Large Deformation	On
	Numerical Damping	0
	Time Step	10^−4^ s
FLUENT	Solver	Transient, Pressure-Based, Absolut Velocity Formulation
	Model	Viscous (Laminar)
	Material: Air	Ideal Gas (FLUENT Database)
	Material: IPA	Compressible Liquid (FLUENT Database)
	Dynamic Mesh	Smoothing (Spring-Based), Remeshing 2.5D, Implicit Update
	Solution Stabilization	Coefficient-Based = 10^−3^
	Solution Methods	SIMPLE
	Spatial Discretization	Gradient: Least Squares Cell-based Pressure: PRESTO!
		Momentum: Second Order Upwind
		Transient Formulation: Second Order-Implicit
	Solution Controls	Default
	Residuals/Convergence-Criteria (Absolute)	Continuity, X-,Y-Velocity = 10^−3^
	Initialization	Standard (Pressure, Velocity) = 0
	Iterations/Coupling Step	5
System Coupling	Min. Coupling Steps/Time Step	5
	End Time	10^−1^ s
	Time Step	10^−4^ s
	Data Transfer/RMS-Convergence Target	10^−2^
	Under Relaxation Factor	1
	Ramping	None
	Execution. Control Sequence	Mechanical = 1, FLUENT = 2

## Appendix B

### Error Analysis and Case Validation

To demonstrate the validity of the simulation results, an additional error analysis was carried out. Major sources of errors in a coupled FSI simulation case, using finite element and finite volume methods, can be improper physical model assumptions as well as discretization errors of the physical equations in spatial and temporal domain. Since the governing equations were solved by the ANSYS Workbench software on a user-defined mesh (e.g., a grid), the stability of the output parameters on the density of the mesh was investigated. The dependence of the spatial discretization in the fluid and structural domain was calculated for a static simulation case of the meshed geometry, as described in Section 2.5. The results, shown in Figure A1, validate a convergence of the output parameter (structural domain: beam displacement, fluid domain: flow rate) for a continuous grid refinement. This indicates that the setup model is stable with respect to the spatial discretization and that there is only a small relative error. A refinement of the time step of one order of magnitude showed no difference on the obtained output parameters of generated pressure and flow rate in the transient simulation case.

A simple case validation was done by an analytical calculation of the expected flow rate in the model geometry. Therefore, the Hagen-Poiseuille equation for an infinite parallel plate channel geometry [23] given by:

(A1) $$Q_{1Phase}=\frac{hw^{3}}{12\eta L}\Delta P,$$

Here, h is the height of the channel, *w* the width, L the channel length, and η the dynamic viscosity of the fluid. This is similar to the 2.5D slice model setup in the ANSYS Workbench and is a valid assumption, considering the aspect ratio. Assuming that the largest pressure drop may occur over the passive valve, the resulting flow will be determined by the channel formed by the open valve and valve seat. In order to calculate the maximum pressure generated by the actuator on the fluid, Equation (6) has been used. A lower value of ΔP=5 kPa was chosen for case validation. Assuming the pressure as a linear varying load over the passive valve beam results in a maximum valve tip deflection of δ=1.5 µm. Together with the initial valve body—valve seat separation of 3 µm, this leads to the following dimensions: w=4.5 µm, h=75 µm, and $L_{valve}=300\;\text{µ}m\;$(the length of the long flow channel through the open valve). The calculation using Equation (9) gives a flow rate of$\;Q_{1Phase}=0.031\;\mathrm{sccm}$ for air, and $Q_{1Phase}=0.0005\;\mathrm{sccm}$ for IPA. This result is in agreement with the simulation result of$\;\;Q_{1Phase}\left(5\;\mathrm{kPa}\right)=0.053\;\mathrm{sccm}$ and$\;Q_{1Phase}\left(5\;\mathrm{kPa}\right)=0.00075\;\mathrm{sccm}$, respectively.
